# Use of a Health Monitoring System During a US Military Exercise During the COVID-19 Pandemic (April 2021): Participant Characteristics, Demographics and Differences in Participation

**Published:** 2024-01-14

**Authors:** Timothy Dignam, Katherine Vandebelt

**Affiliations:** 1 165thAirlift Wing, Georgia Air National Guard; 2 Oracle Corporation

## Introduction

From April 8–24, 2021, approximately 1300 US Air Force and Air National Guard (ANG) military members from 12 wings and support staff participated in a joint US Military air exercise in the southeast US. The coronavirus 2019 (COVID-19) Delta variant was being reported in the United States during this time (1) Because of crowding and close working environments, military personnel are subject to respiratory disease exposure, often greater than in the civilian sector (2). To mitigate potential COVID-19 infections, primarily due to anticipated large gatherings and crowded indoor work settings, leadership requested the development of a COVID-19 health monitoring surveillance system prior to the exercise. Because Oracle America Inc. (Austin, Texas) previously developed a vaccine safety monitoring system with the Centers for Disease Control and Prevention (CDC),(3,4) the ANG requested technical collaboration with Oracle. To conduct symptom surveillance among a healthy population, a custom mobile phone-based, daily health survey, based on requirements from the ANG, was developed by Oracle prior to the exercise and deployed in the Oracle COVID-19 patient monitoring system (PMS) environment using Short Message Service (SMS) reminders. SMS reminders have been effectively used to increase compliance with vaccination schedules, (5) health promotion (6) and improved attendance rates at outpatient clinics and health promotion centers (7,8). The system we employed relied on symptom monitoring. This contrasts with previous studies monitoring military populations, which relied on testing for SARS-CoV-2, the virus that causes COVID-19, for surveillance activities (9,10). The goals of this project were to: 1) provide monitoring and clinical support to military members participating in the mass gathering event who had a known COVID-19 exposure or developed COVID-19 symptoms before, during or after the exercise; 2) quickly provide data required to conduct contact tracing for close contacts of military members who have tested positive for COVID-19; 3) support a safe working environment for military members; and 4) keep leadership updated of COVID-19 occurrence via a dashboard. This report describes the health monitoring system, COVID-19 outcomes, differences in system participation and implications for future military exercises.

## Methods

Units accomplished COVID-19 illness pre-screening, including symptom monitoring and testing of military members before departure to the exercise at the discretion of the home wing. Military members testing negative for SARS-CoV-2 before departure were allowed to join the exercise. More than 500 military members comprising four wings and support staff worked, lodged, and dined at the primary exercise base, which included air and ground training facilities. The remaining approximately 800 military members and other wings participated in the exercise at other sites across the southeast US. The exercise director assembled a five-member COVID-19 public health/medical team consisting of a medic, public health technician, public health officer, nurse, and physician to provide guidance regarding infection prevention, testing, quarantine, isolation, return to work, contact tracing and surveillance.

### Health monitoring

**Figure 1:**
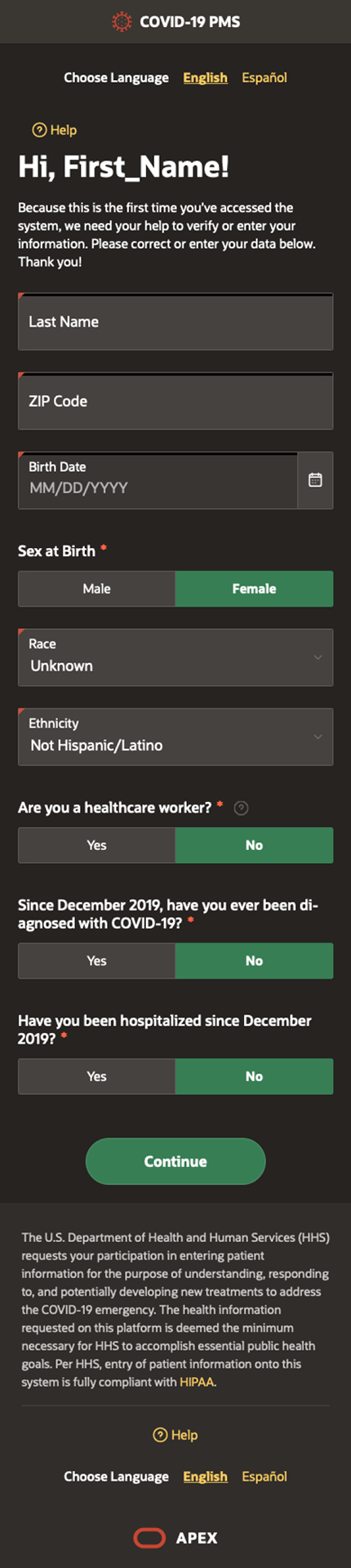


**Figure 2:**
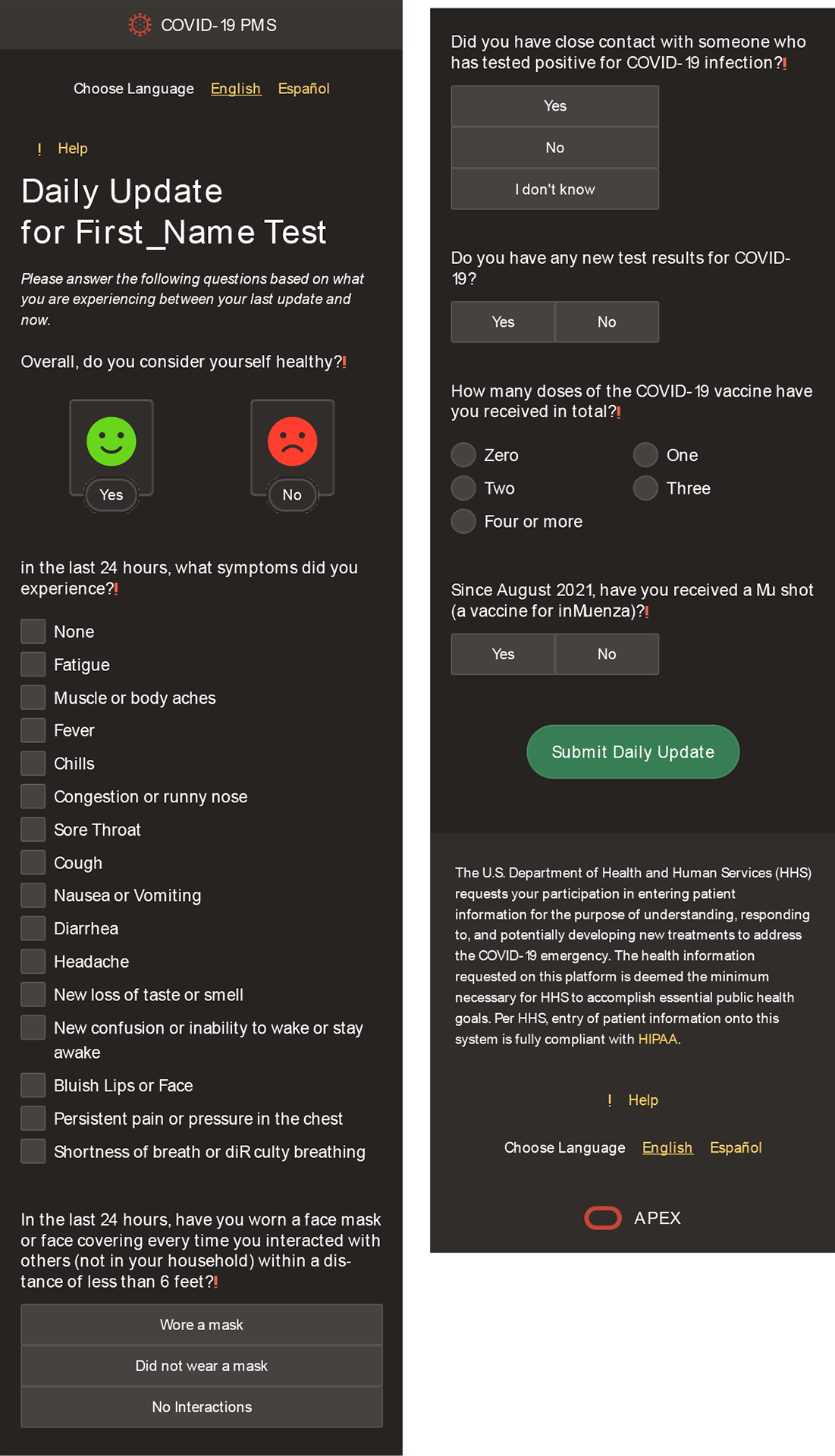


All military member exercise participants were presumed to have mobile phones because 97% of Americans are estimated to own a cell phone (11). The web-based, user-friendly, smartphone SMS health monitoring system was used daily from April 12 to 21 (or 10 days when all military members arrived at the exercise) and allowed the public health/medical team and leaders (i.e., command staff and First Sergeants) from each wing to communicate and track military members who were reporting sick or exposed to COVID-19 positive people. The system included a dashboard for leadership to view real-time aggregate metrics. Leadership was not able to view individual- level health data. The team focused on symptom monitoring of military members from wings and support staff participating at the primary exercise location. All military members who participated in the exercise were eligible for inclusion in data analyses. Military members had their name, birthdate, mobile phone number and ZIP code collected pre-exercise by First Sergeants. Upon exercise arrival, most military members received a welcome brief about COVID-19 risks and prevention and an overview of the health monitoring system. They were sent a text message from Oracle COVID-19 PMS system to validate their information and participate in the system (Figure 1). A small number of military members missed the welcome brief because of travel delays. Participation in health monitoring was strongly encouraged but not mandatory. Military members received a message requesting their participation when they joined the symptom monitoring and to enter their daily health information: The U.S. Department of Health and Human Services and this exercise thank you for your participation in entering information for the purpose of understanding, responding to, and potentially developing new treatments to address the COVID-19 emergency’ (Figure 1). After participants voluntarily opted into the system and validated their information, the medic and public health technician added participants to the system one by one or in bulk. After participants were entered, they received a text message. After receiving the message, participants could start participating immediately in the daily health update survey. Military members were defined as participating in the health monitoring system if they validated their identifying information during the exercise. Validation required the member to consent to participate and correctly enter their last name, date of birth and ZIP code via their mobile phone. Entries were validated against the information provided by First Sergeants pre-exercise. Military members were locked out after three failed validation attempts. Following validation, the military member was prompted to answer seven questions (Figure 2).

**Figure 3:**
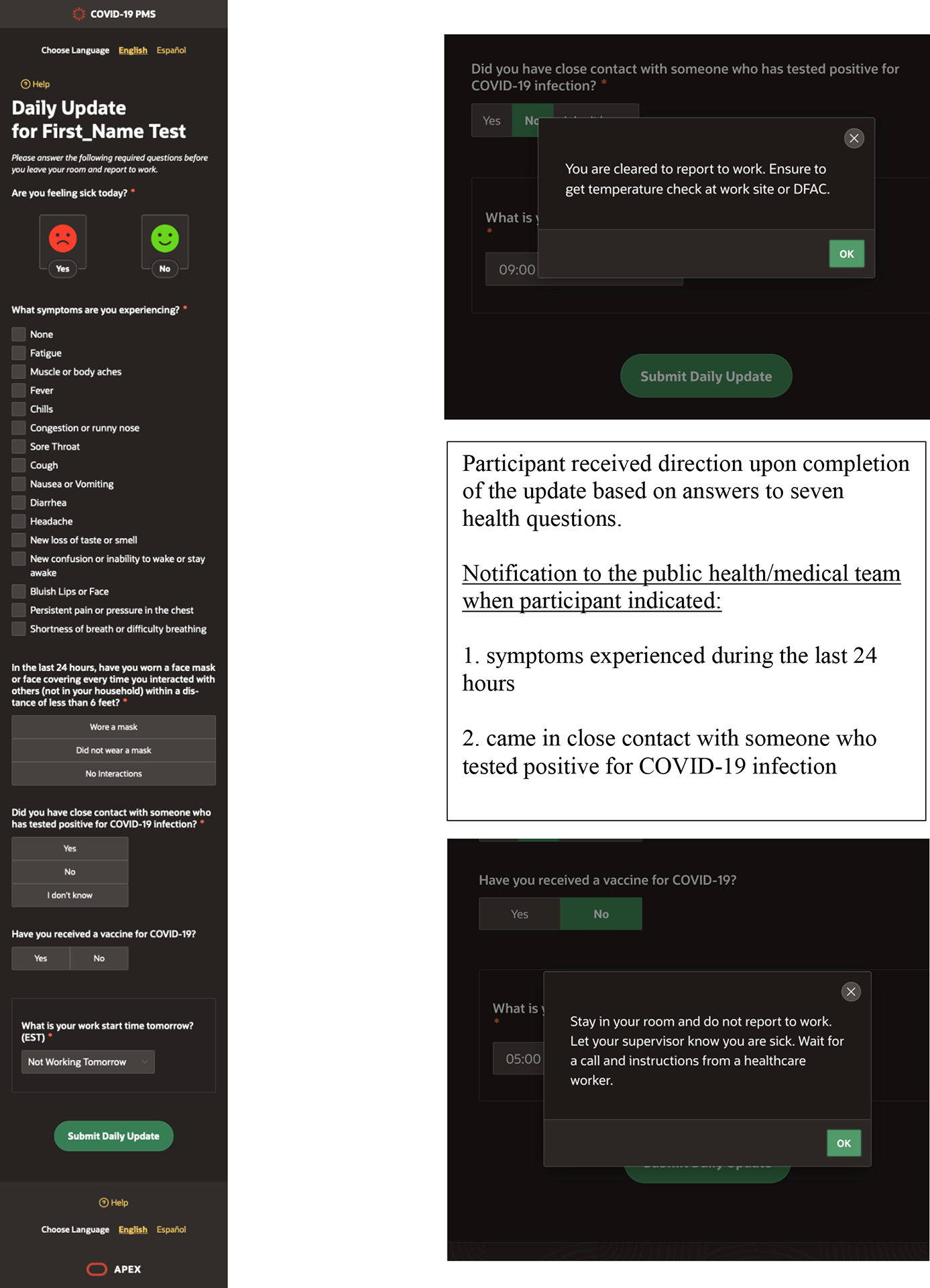


A daily individualized text message web link was sent to each participant on subsequent days, displaying six questions about general wellness, symptoms, behaviors, and potential COVID-19 exposures (Figure 3). If the military member reported feeling ill, they were prompted to notate their symptoms using a checkbox. Symptoms included the following: fever or chills, cough, shortness of breath or difficulty breathing, fatigue, muscle or body aches, headache, new loss of taste or smell, sore throat, congestion or runny nose, nausea or vomiting and diarrhea (12). Military members who reported symptoms daily were aggregated and discussed among the COVID-19 public health/medical team and wing First Sergeants to determine if testing or other actions were required. Because work shifts varied, military members were requested to state their starting time for the next day via the system. The system used the start time information to send a text message 2 hours prior to the work shift or at 0800 if the participants stated they had the day off. Monitoring continued 3 days post-exercise to ensure military members’ health was monitored upon arrival home. When participants self-reported being ill via the health monitoring system, the public health/medical team received a text message and/or an email notification about the member. Military members were informed by the system to inform their supervisor and to stay in their room until further notice. This allowed the team to act swiftly and initiate the appropriate protocol. Military members who did not report their daily status were sent system reminder(s) by the tool to complete the self-assessment. Oracle America Inc. provided technical support during the exercise. Oracle America Inc. and the host ANG wing signed a memorandum of agreement to formalize this government-private sector partnership.

### SARS-CoV-2 testing

On site, COVID-19 polymerase chain reaction (PCR) testing test kits were available from the public health/medical team and offered to all military members. Testing supplies consisted of Remel Microtest M4RT, no beads (Thermo Fisher) and sterile flock nasal swabs (Puritan Medical Products). Tests were transported and analyzed at a nearby US Air Force base laboratory using GeneXpert for nucleic acid testing (Cepheid, Sunnyvale, CA, USA). COVID-19 results were available within 24 hours via the Armed Forces Health Longitudinal Technology Application (AHLTA). Healthcare workers wore personal protective equipment (i.e., gloves, face shields, masks, and gowns) to test military members for possible COVID-19 infection.

### Mask wearing

Per US Department of Defense (DOD) guidance at the time of the exercise, mask wearing was mandatory during participation in the exercise (unless it interfered with the mission) (13).

### Temperature self-check stations

At five locations throughout the flight line were self-check, forehead thermometers (BERRCOM^®^ non- contact digital infrared thermometer), hand sanitizer and daily sign-in sheets. The sign-in sheets allowed people to note their temperature and validate that they had no COVID-19 signs or symptoms as defined by CDC in April 2021 (12) before reporting to work site.

### Return to work

Military members who answered during their daily update: 1) to feeling ill; 2) recording one or more symptoms; or 3) reporting close contact with someone who tested positive for COVID-19, received a notification not to report to work, to let their supervisor know they were sick or exposed and that a public health/medical team member would be calling them. The system sent a text and or an email notification to the medical/public health team of the military members who reported sick. The team followed up with all military members and initiated the appropriate testing, contact tracing, isolation, and quarantine protocols. Military members who did not report their daily status were sent system reminders by the tool to respond to the daily symptom monitoring.

### Data

We examined three sources of data: 1) Air Force medical records; 2) Oracle COVID-19 health monitoring database; and 3) a participant return to work and contact tracing tracking database. We extracted military member demographic, Air Force career field, unit, rank, COVID-19 immunization status, medical readiness and home unit data from ANG medical records. Data regarding previous COVID-19 illness and hospitalization, and healthcare worker status were collected from participants in the health monitoring system. The tracking database was managed by the public health/medical team. It included all documentation of participants reporting symptoms and close contacts of confirmed COVID-19 cases, presumptive cases and/or cases that have sought testing in the absence of symptoms. Wing names were removed and assigned letters A, B, C and D during data analyses. The de-identified, limited analytic data are available from the corresponding author upon reasonable request.

### Vaccination status

Military members were vaccinated against COVID-19 disease per DOD guidance as of April 2021 (14). Military member exercise participant vaccination status was calculated based on their pre-exercise status.

### Statistical analysis

Military member personal information was removed, and a de-identified analytic database was created. Descriptive data analyses of sample distributions and characteristics of those invited to participate in health monitoring were accomplished. Military member demographic characteristics and personnel information were analyzed to predict participation in the system. The number of military members who started and ended participation before the exercise ended (‘drop out proportion’) was also calculated. Bivariable and logistic regression analyses were conducted to assess military member characteristics associated with participation in the health monitoring system. Characteristics significantly associated (P < 0.05) with system participation were evaluated in multivariable analyses. Multivariable analyses assessed each characteristic one at a time. Statistically significant characteristics (P < 0.10) identified in the first multivariable analysis were included in a second multivariable analysis. During the second multivariable analysis, we used a forward- selection strategy to add one characteristic at a time to the most predictive model until all characteristics in the model were statistically significant (P <.05). Interactions between characteristics and the confounding variable were assessed. Variance inflation factors were used to assess collinearity between variables in predictive models. Data analyses were conducted using SAS version 9.3 (SAS Institute Inc., Cary, North Carolina).

### Security

Oracle Inc. developed personal security measures in partnership with DOD and provided the secure, protected platform used during the exercise. Oracle America Inc. and the host Air National Guard wing signed a memorandum of agreement to use the COVID-19 health monitoring system. Oracle America Inc. implemented and maintained appropriate technical and organizational security measures for processing personal information to prevent accidental or unlawful destruction, loss, alteration, or unauthorized disclosure of personal information. These security measures govern all security areas applicable to the health monitoring platform, including physical access, system access, data access, transmission and encryption, input, data backup, data segregation and security oversight, enforcement and other security controls and measures.

### Human subjects protection

The Department of the Air Force, Component Office of Human Research Protections reviewed the study protocol. The activity was determined not to be human subject research and was exempt from the human subject internal review board.

## Results

### Military member characteristics

Five hundred and twenty-nine military members were invited to participate in COVID-19 health monitoring, and 419 (79%) participated. The average military member age among invited participants was 34.2 years (min./max.: 19–64 years). One hundred and four invited military members (20%) were 25–29 years of age; 441 (83%) were male; 437 (83%) were enlisted; 330 (62%) were fully or partially vaccinated; 444 (84%) were medically ready to deploy; and most military members (96%) were not health care workers (Table 1). Ninety-two (92) participants were officers, and 71 (77%) were classified as either pilots/flyers/ flyer instructors or operations.

### Military member participation characteristics

Among the 419 military members who validated their personal information, they did so, on average, within one day (min./max.: < 1–5 days, std. dev.: 1.3 days), and 398 (95%) did so within 3 days. System participants were more likely to be enlisted (n=375, 89%) from Airlift Wing A (n=l37, 32%) and Airlift Wing B (n=137, 32%), male (n=344, 82%), ages 19–39 years (n=317, 76%), fully vaccinated against COVID-19 (n=206, 49%), medically ready to deploy (n=352, 84%), and in the logistics and maintenance career field (n=304, 73%) (Table 1). The most common subgroups who validated and participated in health monitoring were those who were medical personnel (100%), from Wing A (99%). partially vaccinated (96%). ages 19–24 years (91%), in the logistics and maintenance career field (90%), enlisted (86%) and female (85%). The dropout rate using the system was two military members (0.2%).

### Predictive factors

In bivariable analyses, non-participation in the system was independently significantly associated with being a member of any wing or support staff other than Wing A (OR 53.0, 95% CI 7.3, 383.2), being fully vaccinated for COVID-19 (OR 8.9, 95% CI 2. 1, 37. 5) or not started the COVID-19 vaccination process (OR 8.5, 95% CI 2.0, 36.7), being an officer (OR 6.6, 95% CI 4.0, 10.8), being in a career field other than logistics and maintenance (OR 5.9, 95% CI 3.7, 9.3), being in any age group except 19–24 years (OR 3.2, 95% CI 1.4, 7.1) (Table 1). Based on multivariable analyses, non-participation in the system was significantly associated with being a member of any wing or support staff other than Wing A (OR 43.3, 95% CI 5.9, 318.4), being an officer (OR 3.5, 95% CI 1.9, 6.3), and being in a career field other than logistics and maintenance (OR 2.8, 95% CI 1.6, 4.8) (Table 2). Age (considered both as continuous and as age groups) was not significant in the multivariable model and did not strengthen the final model (i.e., lower the Akaike information criterion). Officers who were invited to participate in the military exercise were, on average, older (avg 39 years) compared with invited enlisted members (avg 33 years) (P < 0.0001). Collinearity assessment did not identify significant correlations between variables in the models.

### Cases, contact tracing and vaccination

The public health/medical team tracked 4666 daily status updates. Which, among 419 participants, yields 11.1 status updates/participant (or, on average 1.1 daily status updates for each participant during the 10-day monitoring period). Twenty-seven military members (6%) reported various symptoms during the exercise. The most frequently reported symptoms were sore throat (n=8), headache (n=7), muscle or body aches (n=6), congestion or runny nose (n=6), and fatigue (n=6). All military members who reported symptoms had follow-up provided by First Sergeants, flight doctors and/ or the public health/ medical team. Many symptoms were determined to be from allergies and post-COVID-19 vaccine administration side effects. The COVID-19 public health/medical team administered 14 initial and return-to-work COVID-19 tests to seven military members and tracked 11 with known COVID-19 close contact exposure (i.e., within 6 feet, for at least 15 minutes, over a 24-hour period) (15).

Three exposed were asymptomatic, fully vaccinated and returned to work. Six military members were quarantined, tested negative, were asymptomatic and returned to work on post-exposure day 8. The remaining military member tested positive, was isolated in an assigned room, instructed to end participation in the exercise, and was sent home via personal vehicle to further isolate for 14 days. Fifty-five military members received a first or second dose of Moderna COVID-19 vaccinations during the exercise (administered by the local ANG medical group) (16).

## Discussion

Several COVID-19 health and symptom monitoring systems have been used during the COVID-19 pandemic (17–20), but predictors of participation using such systems have been minimally explored. We did not find prior studies about COVID-19 health or symptom monitoring among military populations. Previous civilian reports show participation or intention to participate percentages lower than we observed during our military exercise. Meyer and colleagues reported daily COVID-19 symptom monitoring using a mobile phone app questionnaire among male German professional football players and game officials (17). They found that 64% of the players and 47% of the officials participated in the system over a 9-week period (May-July 2020). However, reasons for differences in participation were not explored. Dutch researchers examined predictors of intention to use a COVID-19 mobile phone symptom monitoring app among 238 adults (21). They found that 45% of respondents were willing to use a mobile application for COVID-19 symptom recognition and monitoring and that younger age, attitude towards technology and fear of COVID-19 were predictors of intention to use. Another study examined attitudes towards using a mobile phone app or a website to track their COVID-19 symptoms and receive recommendations (22). The study conducted April- June 2020 among 10 760 US adults with chronic health conditions reported that 22% of respondents were extremely/very likely to use a mobile phone app or a website to track their COVID-19 symptoms.

Most military members (79%) invited to participate in our health monitoring system registered and reported daily health status updates. We identified three factors that predicted participation in health monitoring: being enlisted, being a member of Wing A and being in the logistics and maintenance career field. A potential reason for lower participation among officers was that 26/27 (96%) of the pilots/ flyers were officers. In bivariable analysis, pilots/ flyers were 8.3 times less likely (95% CI 3.6, 19.1) to participate in the system compared with logistics and maintenance career fields. Perhaps the attitude of aircrew towards medical monitoring (i.e., increased risk of non-flying status) created the participation difference that was observed between officers and enlisted members. Similarly, operations officers were 7.3 times more likely (95% CI 4.3, 12.4) to not participate in the health monitoring system. In a 2019 study, Britt and colleagues examined barriers and facilitators of treatment-seeking for mental and physical health problems among a US Military population (23). They found that more officers preferred managing mental and physical health problems independently compared to enlisted personnel. This “do it yourself” attitude provides a potential reason for lower participation among officers in the present study. A final potential reason for lower officer participation was decreased officer attendance at pre-exercise briefings. Several pre-exercise briefings were conducted, which provided an opportunity to review the usage of the COVID-19 health monitoring system and the enrolment process. Missing attendance at these briefings may have decreased awareness about symptom monitoring and lack of participation. However, we did not collect attendance information at pre-exercise briefings; this is an observational finding.

Members from Wing A demonstrated significantly higher participation in health monitoring compared to other wings and support staff. A potential reason for high compliance was Wing A had a very proactive First Sergeant who ensured full attendance at the Wing A pre-exercise briefing, strongly encouraged participation in the system and was available for technical assistance. For example, the First Sergeant from Wing A corrected errors with member phone numbers and quickly reported technical issues for resolution by the public health/medical team.

Members in the logistics and maintenance career field were significantly more likely to participate in health monitoring during the exercise. The reasons for this finding are unclear. A possible reason is that 40% of members in the logistics and maintenance career field were younger (i.e., 29 years) and most (98%) were enlisted, a predictor of participation.

Military member attendance at pre-exercise briefings appears to be important to health monitoring participation. Military members were educated about receiving a text message (i.e., not spam). instructions to enroll, where to report if ill, and received guidance to stay safe during the exercise. Personnel briefed at the in-processing briefings were notified that the COVID-19 public health/medical team would be on call 24/7 for questions regarding guidance with symptoms or potential exposure to COVID-19. If injuries or illnesses were life-threatening, members were instructed to report to a local hospital for care. During pre-exercise briefings, members were informed of the importance of self-assessment and reporting daily health status to have a successful exercise.

During the exercise, only one military member was confirmed positive via PCR testing for COVID-19 illness. The public health/medical team played an essential role in overseeing the health of the military members participating in Air Force and ANG military exercises. Communication, preparation, protocols, health monitoring and vaccination were the team’s keys to successful health management. Protocols from the CDC and Georgia State Health Department were reviewed by the public health/ medical team with visiting Wing physicians and the Georgia State Air Surgeon. Agreement was reached on return-to-work guidance, masking guidance and close contacts exposure guidelines for quarantine to ensure that the public health/medical team provided current information to inform pre-exercise briefings, COVID-19 health monitoring system requirements, leadership, and public health on-site operations.

Access to military member health records and daily health status provided the COVID-19 public health/ medical team adequate information to meet real- world responsibilities: 1) monitoring and clinical support to military members; 2) quickly providing data required to conduct contact tracing for close contacts of military members who have tested positive for COVID-19; 3) support a safe working environment for military members; and 4) update leadership about suspected/ confirmed COVID-19 occurrence via a dashboard.

Being fully vaccinated had a positive impact on operational success. Per exercise protocol, vaccinated, asymptomatic military members with known exposure from a COVID-19-positive case reported back to work. Military members who had COVID-19 illness within the previous 3 months, recovered and remained without COVID-19 symptoms were to return to work, per protocol. Upon arrival, 61% of participants were fully or partially vaccinated. The COVID-19 health monitoring system supported a safe work environment, enhanced by the temperature check stations and mask-wearing guidelines.

This is the first study we are aware of that reports on using mobile technology for self-reported symptom health monitoring/reporting among a military population. The findings have broader implications for future military exercises, military readiness, and digital health environments. Exercise training time is valuable and can be expensive. We provided easily available and rapidly deployable mobile technology, allowing leadership to focus on executing the exercise mission rather than the potential disruption due to widespread respiratory disease exposure. Regular information sharing with leadership, using a small health team to address potential health issues, user- friendly technology, pre-planning and agreement on return-to-work health protocols may be helpful in future military events. Our symptom monitoring system is potentially useful for identifying disease trends and possible cases during future military exercises. Symptom monitoring information could be combined with self-reported vital signs data, geographic information, and ‘telehealth’ to address emerging health issues rapidly in the military setting. Soldier acceptance and provision of actionable information to leadership are important factors to ensure success for future military exercises.

This study is subject to limitations. We did not survey non-participants about why they did not participate in the health monitoring system (e.g., concern regarding cost of receiving text or data security). Identifying barriers to participation is an area of future research. Repeated PCR testing represents the current ‘gold standard’ for assessing COVID-19 diagnosis. Symptom reporting is a weaker indicator of the presence of COVID-19 disease, especially among younger populations.^24^ However, our experience was that among our younger population (average age 34 years), symptom monitoring with daily temperature checks and education about prevention proved proactive in preventing COVID-19. Although not designed into the health monitoring system, a potentially helpful system capability is to broadcast health messages during the event. Examples of such messages include building closures, COVID-19 prevention measures and health-related updates from leadership.

## Conclusion

Today, mobile phones and apps are ubiquitous. During a public health emergency, the use of a web- based, smartphone health monitoring system was an opportunity to collect real-time health monitoring data and provide a strategy to facilitate and establish procedures for a safe return to normal operations. Most exercise members self-assessed and reported daily health status updates via the health monitoring system. We found significantly higher COVID-19 health monitoring system participation among the enlisted, members from Wing A, and logistics and maintenance personnel. In the future, military leaders may consider mandatory attendance at in-processing briefings or use technology to video link military members. Special attention should also be given to certain Air Force career fields. Future studies could be conducted to determine barriers to officer participation in health monitoring system usage. Health monitoring allowed exercise leadership to focus on mission command and control. Health monitoring allowed for early recognition of symptoms associated with respiratory illness, where an outbreak would have disrupted an important military exercise.

## Supplementary Material

supplemental figure 1

## Figures and Tables

**Table 1:** Member characteristics (N=529)

Member characteristic	Number (%)	Member participation in system (%)	Non-participation odds ratio (95% confidence interval)
**Health monitoring system participation**			
Participated	419 (79.2)	NA	NA
Never participated	110 (20.8)		
**Air Guard Wing/grouping**			
Wing A	138 (26.1)	137 (99.3)	Ref
Wing B	177 (33.5)	137 (77.4)	40.0 (5.4, 295.0)
Wing C	128 (24.2)	90 (70.3)	57.8 (7.8, 428.8)
Wing D	55 (10.3)	36 (65.4)	72.3 (9.4, 558.3)
Exercise support staff	31 (5.9)	19 (61.3)	86.5 (10.6, 703.4)
All Wings/support staff versus Wing A	391 (73.9)	282 (72.1)	53.0 (7.3, 383.2)
**COVID-19 Vaccination status**			
Full	274 (51.9)	206 (75.2)	8.9 (2.1, 37.5)
Partial	56 (10.6)	54 (96.4)	Ref
Not started	134 (25.3)	102 (76.1)	8.5 (2.0, 36.7)
Medical decline	65 (12.2)	57 (87.7)	3.8 (0.8, 18.6)
**Individual medical readiness**			
Current/due	444 (83.9)	352 (79.3)	Ref
Overdue	85 (16.1)	67 (78.8)	1.0 (0.6 1.8)
**Personnel classification**			
Enlisted	437 (82.6)	375 (85.8)	Ref
Officer	92 (17.4)	44 (47.8)	6.6 (4.0, 10.8)
**Enlisted and officer career category**			
Commander/director	4 (0.8)	1 (25.0)	26.8 (2.7, 265.0)
Logistics & maintenance	338 (63.8)	304 (89.9)	Ref
Medical	14 (2.6)	14 (100.0)	Undefined*
Operations	100 (18.9)	55 (55.0)	7.3 (4.3, 12.4)
Pilot/flyer/flying instructor	27 (5.1)	14 (51.9)	8.3 (3.6, 19.1)
Special duty	8 (1.6)	8 (100.0)	Undefined
Support	36 (6.8)	21 (58.3)	6.4 (3.0, 13.5)
Weather/meteorological	2 (0.4)	2 (100.0)	Undefined
** **All career categories versus logistics & maintenance	191 (36.1)	115 (60.2)	5.9 (3.7, 9.3)
**Healthcare personnel**			
Yes	22 (4.2)	22 (100.0)	Ref
No	507 (95.8)	397 (78.3)	Undefined
**Gender**			
Male	441 (83.4)	344 (78.0)	1.6 (0.9, 3.1)
Female	88 (16.6)	75 (85.2)	Ref
**Age (years)**			
19–24	81 (15.3)	74 (91.4)	Ref
25–29	104 (19.6)	82 (78.9)	2.8 (1.1 7.0)
30–34	101 (19.0)	81 (80.2)	2.6 (1.04, 6.5)
35–39	103 (19.5)	80 (77.7)	3.0 (1.2, 7.5)
40–44	72 (13.6)	48 (66.7)	5.3 (2.1, 13.2)
45–49	33 (6.2)	23 (69.7)	4.6 (1.6, 13.4)
50+	35 (6.6)	31 (88.6)	1.4 (0.4, 5.0)
All age groups versus 19–24 years	448 (84.7)	345 (77.0)	3.2 (1.4, 7.1)
**Previous COVID-19 diagnosis**			
Yes	52 (9.8)	52 (100.0)	NA
No	368 (69.6)	367 (99.7)	
Missing	109 (20.6)	0 (0.0)	
**Previous COVID-19 hospitalisation**			
Yes	6 (1.2)	6 (100.0)	NA
No	414 (78.2)	413 (99.8)	
Missing	109 (20.6)	0 (0.0)	

* Undefined: Small cell sizes (<5) do not allow the normal approximation odds ratio to be calculated.

**Table 2:** Multi-variable logistic regression estimates of the association between symptom monitoring participation and other member characteristics, (N=529)

Member characteristic	Beta	Non-participation odds ratio (95% confidence interval)	Standard error	Wald chi-square	P-value
**Air Guard Wing**					
Wing A		Ref			
All other wings and support staff	3.77	43.3 (5.9, 318.4)	1.018	13.71	0.0002
**Personnel classification**					
Enlisted		Ref			
Officer	1.25	3.5 (1.9, 6.3)	0.3042	16.84	<0.0001
**Enlisted and Officer career category**					
Logistics & maintenance		Ref			
All other career fields	1.03	2.8 (1.6, 4.8)	0.277	13.89	0.0002

## Data Availability

Limited, deidentified analytic data will be available upon request.
