# Supplementary material for: Use of a Health Monitoring System During a US Military Exercise During the COVID-19 Pandemic (April 2021): Participant Characteristics, Demographics and Differences in Participation
Source: J Mil Veterans Health. Author manuscript; Available in PMC 2024 Aug 23. (PMC11342795)
Supplement: supplemental figure 1 [file NIHMS2009203-supplement-supplemental_figure_1.docx]

**
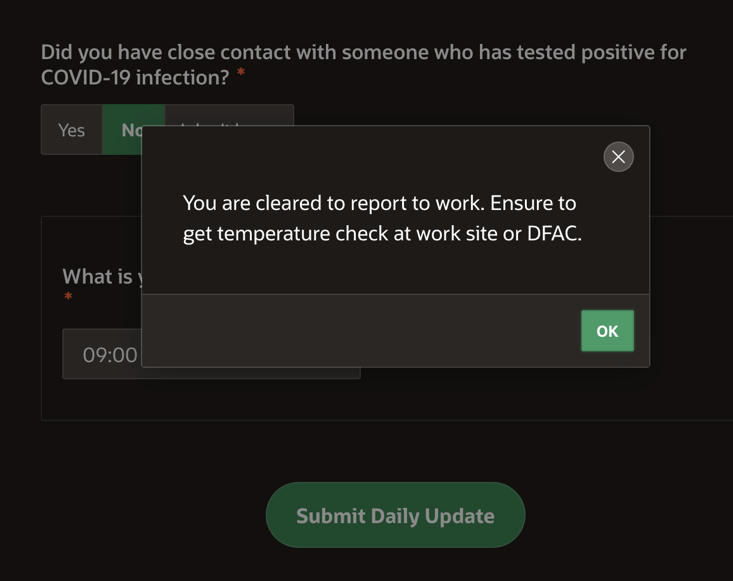

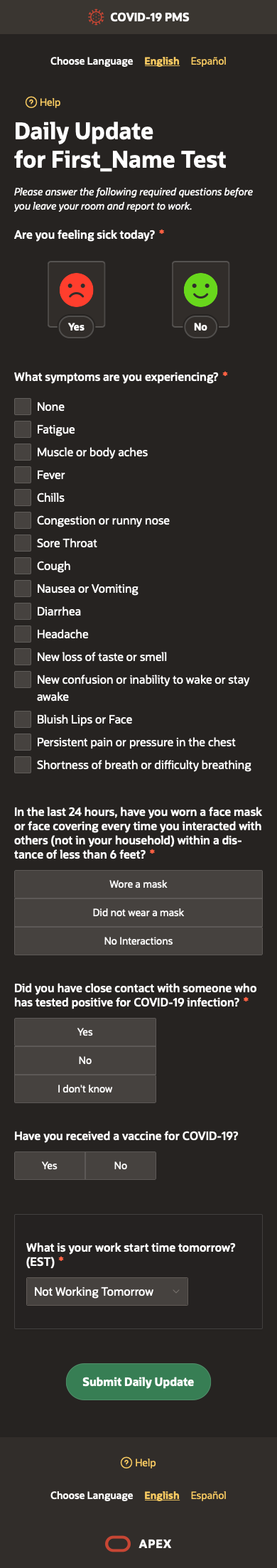

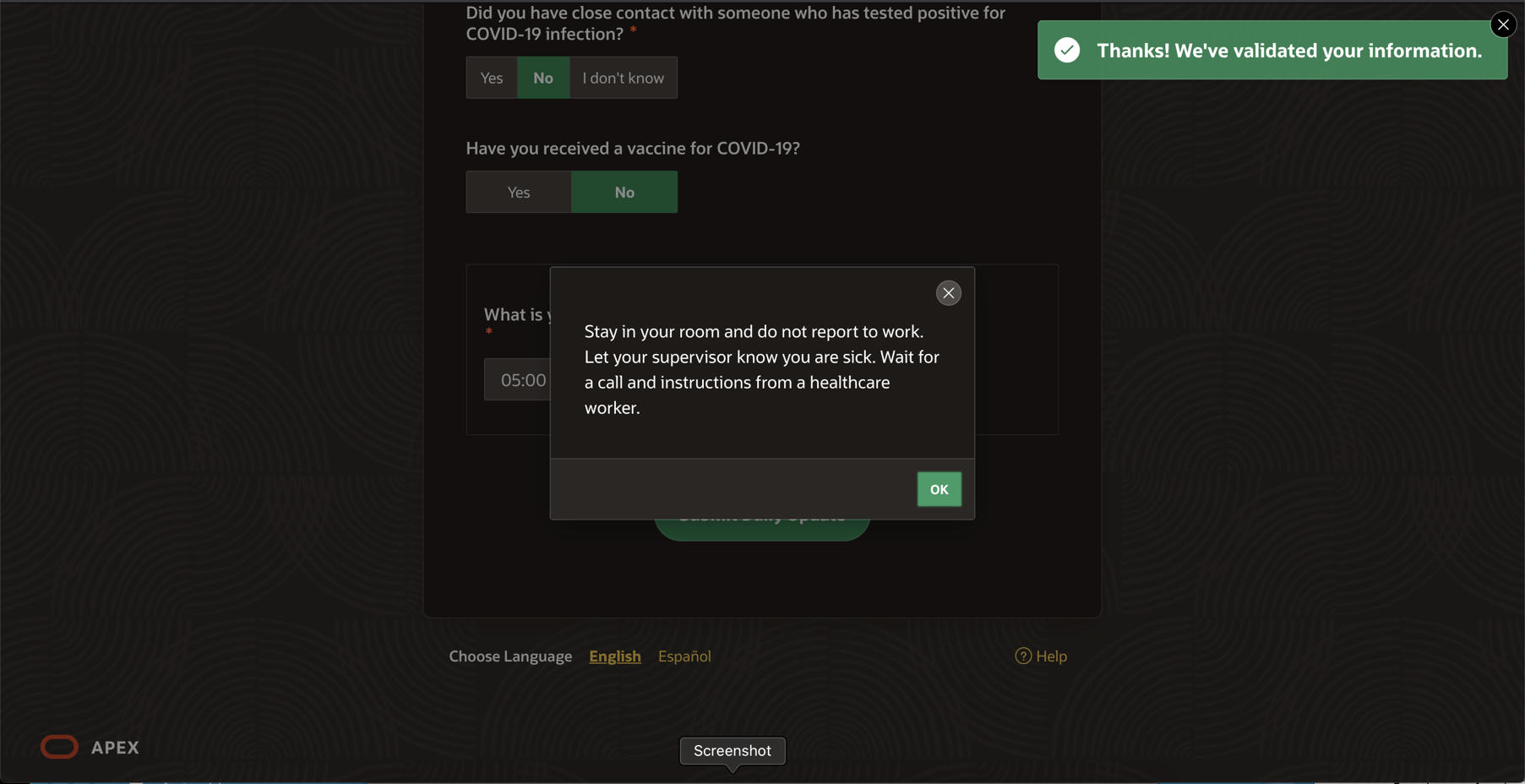
**

**Supplemental Figure 1: Six Daily Health Questions**

Participant received direction upon completion of the update based on answers to questions

Notification to the public health/medical team when participant indicated

1. symptoms experiences during the last 24 hours
2. came in close contact with some tested positive for COVID-19 infection
